# Gradually Enlarging Nodular Pulmonary Amyloidosis Associated With Sjögren's Syndrome

**DOI:** 10.1002/rcr2.70739

**Published:** 2026-08-28

**Authors:** Noboru Morikawa, Satoshi Mikami, Daisuke Suzuki

**Affiliations:** ^1^ Department of Respiratory Medicine Chutoen General Medical Center Shizuoka Japan; ^2^ Department of Diagnostic Pathology Chutoen General Medical Center Shizuoka Japan

**Keywords:** autoimmune diseases, bronchoscopy, pulmonary amyloidosis, Sjögren's syndrome

## Abstract

A 65‐year‐old woman with primary Sjögren's syndrome without a history of immunosuppressive therapy presented with progressively enlarging pulmonary nodules showing calcification and cystic changes, mimicking malignancy. Histological examination confirmed AL λ‐type nodular pulmonary amyloidosis. Recognition of this radiological pattern is important to avoid misdiagnosis and unnecessary invasive investigations.

## Graphical abstract

A woman with Sjögren's syndrome presented with progressively enlarging pulmonary nodules showing calcification and cystic changes, mimicking metastatic malignancy. Histological examination confirmed AL λ‐type nodular pulmonary amyloidosis. Recognition of this radiological pattern is important to avoid misdiagnosis and unnecessary invasive interventions.

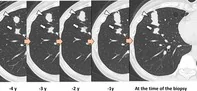

A 65‐year‐old woman with primary Sjögren's syndrome, who had remained clinically stable without immunosuppressive therapy for 10 years, was referred for evaluation of pulmonary nodules detected on serial chest computed tomography (CT). Over 4 years, the largest nodule enlarged from 16 to 36 mm, accompanied by calcification and cystic changes in the surrounding lung parenchyma (Figures 1 and 2), without respiratory symptoms. Tumour markers, interferon‐γ release assay, and serum β‐D‐glucan were unremarkable. Bronchoscopic biopsy using radial endobronchial ultrasound of a right B4b lesion revealed Congo red–positive amorphous eosinophilic deposits. Immunohistochemical staining demonstrated λ light‐chain restriction, confirming AL λ‐type nodular pulmonary amyloidosis (Figure 3). The patient had hypergammaglobulinaemia, but serum and urine protein electrophoresis revealed no monoclonal protein. There was no evidence of cryoglobulinaemia, hypocomplementaemia, or systemic amyloidosis on abdominal fat pads and gastrointestinal biopsies. Nodular pulmonary amyloidosis is a rare manifestation associated with Sjögren's syndrome [1]. This radiological pattern may mimic pulmonary lymphoma, including MALT lymphoma, or metastatic malignancy [1, 2]. Although these findings are not specific, they may raise suspicion for nodular pulmonary amyloidosis and support histological confirmation for definitive diagnosis. As the diagnosis was based on bronchoscopic biopsy, ongoing surveillance for coexistent lymphoma is warranted.

**FIGURE 1:**
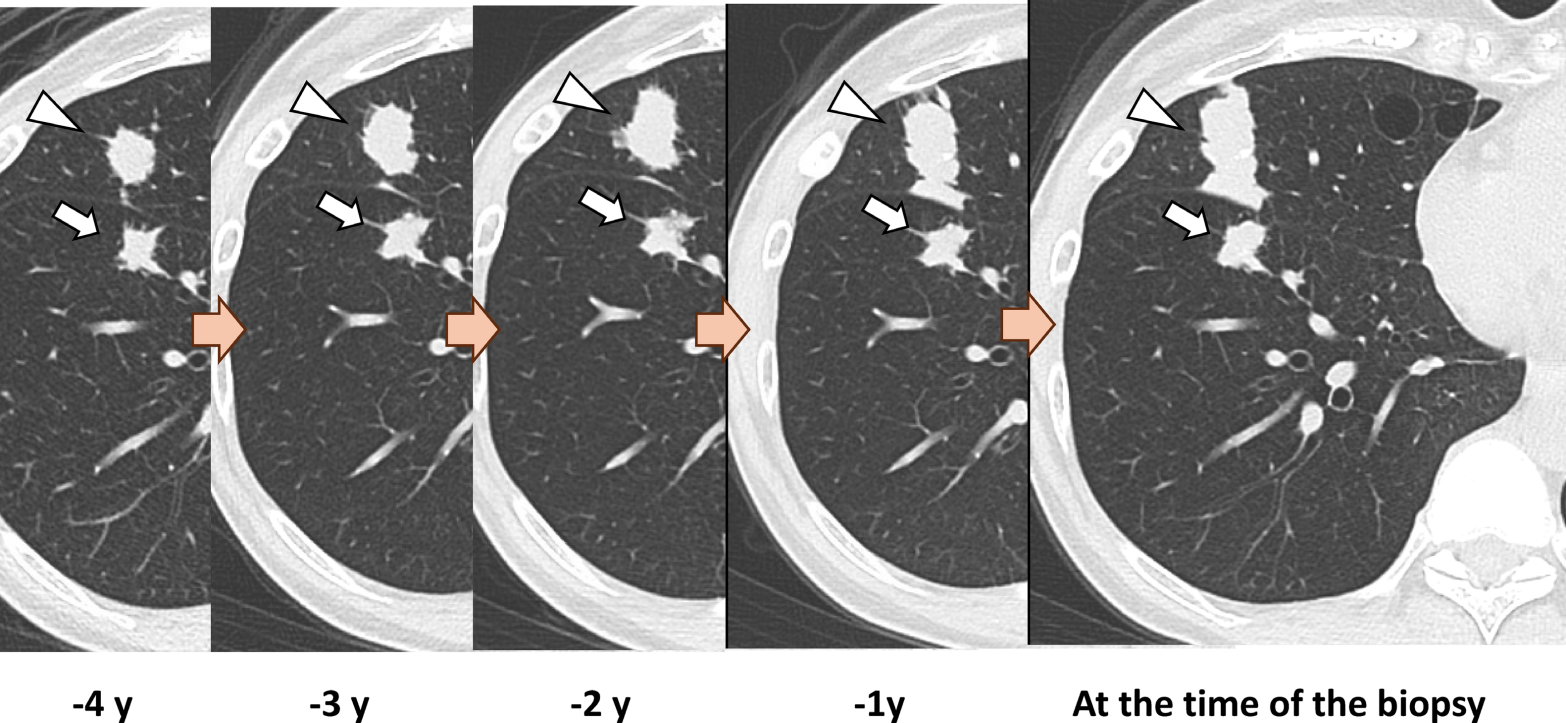
Mass (arrowhead) in the right middle lobe and nodule (white arrow) in the right lower lobe.

**FIGURE 2:**
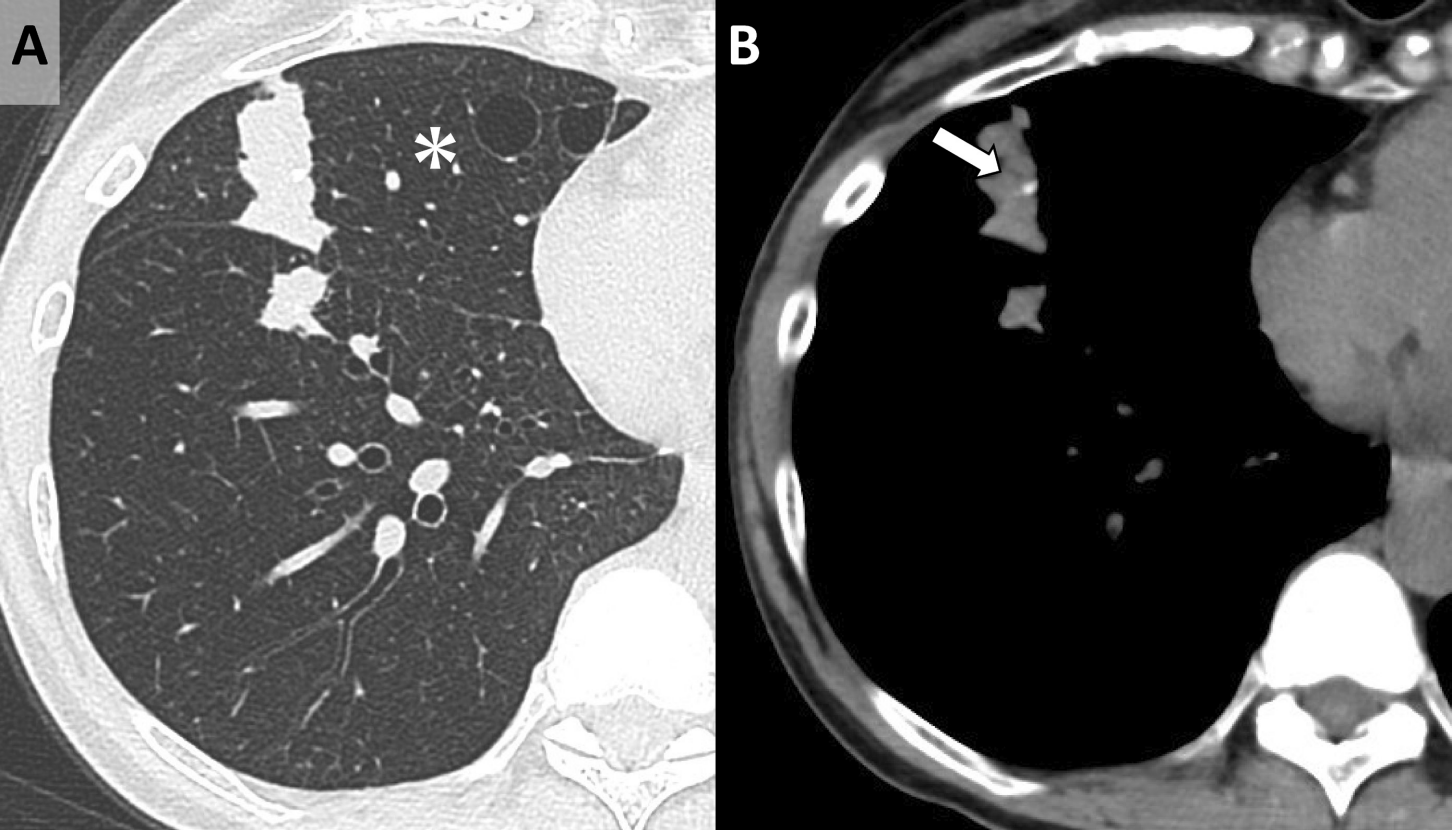
(A) Mass lesions with cystic lesions (asterisk). (B) Calcification (white arrow).

**FIGURE 3:**
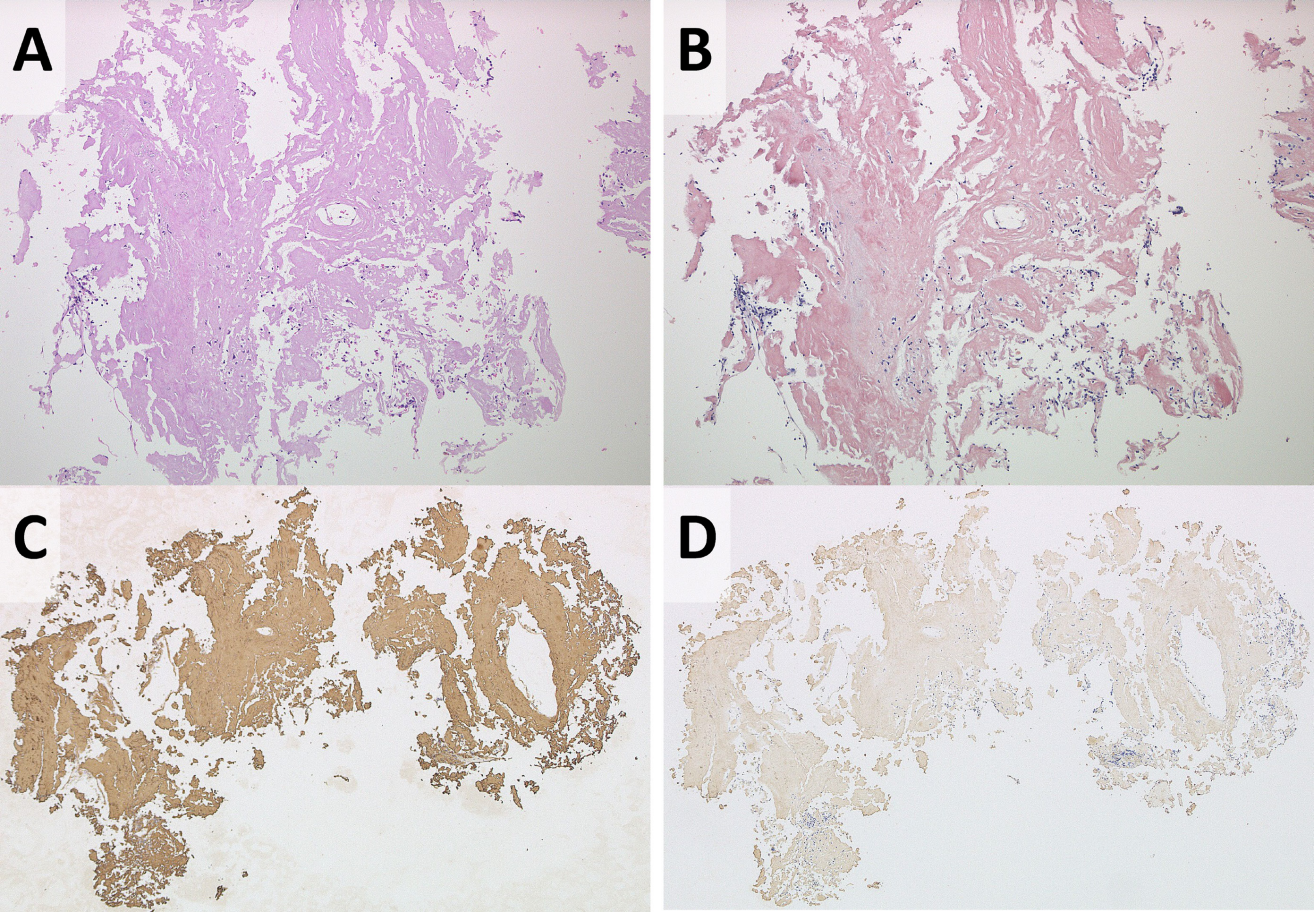
(A) Haematoxylin and eosin staining (×40). (B) Congo red‐staining (×40). (C) Immunohistochemical staining showing positivity for λ light chains (×40). (D) Immunohistochemical staining showing negativity for κ light chains (×40).

## Author Contributions

All authors contributed to the writing, review, and final approval of the manuscript.

## Funding

The authors have nothing to report.

## Ethics Statement

Ethical approval was obtained from the Ethics Committee of Chutoen General Medical Center (approval number: 1348260304; approved date: March 4, 2026).

## Consent

The authors declare that written informed consent was obtained for the publication of this manuscript and accompanying images using the consent form provided by the Journal.

## Conflicts of Interest

The authors declare no conflicts of interest.

## Data Availability

The data that support the findings of this study are available from the corresponding author upon reasonable request.
